# 

**Published:** 1873-04

**Authors:** 


### INDEX TO VOLUME XI.

A

Atlanta Academy of Medicine, 187, 442, 487. 561, 624, 691
Atlanta Daily Herald......544
Atlanta Medical College....234
Atlanta Med. & Surg. Jour. 57
Ala. State Med. Assoc.224, 732
Ark. State Med. Association 599
"A Appeel to the Sextant"..584
Anæsthesia................602
Amaurosis.................386
Abstracts German Journals 92, 205
Agassiz, death of..........606
American Med. Assoc..115, 161
Amer. Jour. Med. Sciences..704
American Jour. Obstetrics..474
American Jour. Pharmacy..526
Amer. Pharmaceutical Asso..351
American Practitioner.....535
Arsenic in asthma.........705
Arsenic in constipation.....494
Arterial action, peristaltic..652
Allingham on the rectum....416
Action moral on physical nature..................455
April number.............. 1
Anti-Opium pill...........591
Aachives of Electrology....608
Aloes in hemorrhoids..... 121
Acology, Westmoreland's..413
Armstrong, W. S., M.D....200
Ackowlegments— 546, 609, 674, 738
Annual Address, Johnson.. 27
Amputation of thigh.......276
August number...........243

B

Battey, Robert, M.D., 1, 65, 224, 261, 323, 428, 478, 479, 483
Baird, J. B., M.D.....187, 553, 561, 622, 624, 675, 691, 698
Barker, Pureperal Diseases.732
Bladder, candle in female...451
Blake, Miss Sophia Jex.....494
Belladonna vs. Opium......240
Bellevue Hospital notes....459
Bibliographical notices....408, 545, 660
Bismuthi, prepa'on liquor..529
Bright's diseases of kidney..675
Biddenden Maids..........725
Bromides.............174, 232
Blood-letting..............220
Bronchitis, chronic.........460
Bloodless amputations...465, 467
Blood transfusion......529, 532
Boston Journal Chemistry...658
Boston Med. & Surg. Jour..579
Boney anchylosis hip joint...653
Burns and scalds..........452
Business notice............479
Burroughs, J. C., M.D.....687
Butler, Dr. S. W., death of...672

C

Cancer, electrolysis in......462
Cancer female breast....462, 470
Cancer, origin and nature— 177, 660
Catarrh female genitals..... 92
Calhoun, A. W., M.D.— 105, 188, 255, 696
Carbolic acid in dysentery....236
Candle in female bladder...451
Caoutchouc electuary......458
Caius, Shakespeare's Dr....495
Castor oil, administration..526
Catheter in hypertrophy of prostate................579
Calculus, expulsion of large uric....................619
Camphorein..............659
Charleston Medical Journal.122
Craving for stimulants, cure.592
Clavicles, spontaneous dislocation.................655

Cerebro-spinal meningitis... 84
Cells and cell life .........419
Cremation club ...........468
Cervical endo-metritis, hypospadias ................587
Certificates misused .......672
Cincho-quinine ........395, 622
Cinchonia, muriate........526
City Hospital.............404
Circumcission in ulcers ....453
Cin; Lancet and Observer..609
Clinic, The........446, 595, 651
Clinic, Atlanta Medical College.......512, 569, 653, 701
Child-mother .........476, 591
Chicago Jour., Nervous and Mental Diseases.........609
Colica Pictonum ..........186
Contributions to Med. Press.228
Correspondence, Editorial..231
Correspondence, Foreign—105, 381, 385, 465
Couvert, A., M.D..........407
Confederate Surgeons, call..418
Constipation, treatment....494
Cod-liver oil, emulsion .....527
Commonwealth, The.......609
Corn-meal a vehicle for heat.658
Columbia Hospital for Women ...................661
Chlorate potassa in carcinoma ...................180
Chloral, action of..........452
Chloral in delirium tremens.586
Chloral in Tetanus ........233
Chloroform, death from....580
Cholera .........205, 237, 287
Choleriac diarrhœa........301
Choking, case of...........406
Croup, treatment..........441
Cholesteræmia ............521
Cutting the throat.........148
Cysts of the iris...........255

D

Daniel Z. T., M.D ..........669
Death rate of cities........690
December number .........483
Delirium tremens..........586
Delivery, hymen intact.....650
Depilatory, new............591
Devotion to science........441
Diabetes mellitus..........187
Dictionary, Dunglison's....663
Dictionary of Elevations...479
Digitalis in delirium tremens.586
Dougherty, Wm. H., M.D.—265, 325
Doctors and drunkards.....644
Doctor's three faces........703
Doctor Caius .............495
Dose and Symptom Book.. 731
Duncan, J. Matthews, M.D. 450, 472, 646
Dunglison's Medic'l Dictionary....................663
Dysentery, carbolic acid in.236
Dysentery, epidemic........476
Dysmenorrhœa............145
Dysmenorrhœa, spasmodic..450

E

Eclampsia, treatment..241, 317
Exmarch's elastic tourniquet...............465, 467
Electricity, Reynolds on ...731
Electrolysis in cancer......462
Electro-surgery, Rockwell & Beard .................607
Electro-therapeutics .......123
Encephaloid of knee.......276
Eve, Paul F., M.D.....381, 383
Excretions, lead in.........506
Ether, administration..581, 602
Ether, death from..........658
Ether, inflammability ......594
Epistaxis, treatment.......241
Epidemics, laws of.........401
Epilepsy in neonatus.......472
Epilepsy, treatment of obstinate.................640
Edinburgh Medical Journal.646
Ergot....................380
Ergot in cholera...........183
Esophagus obstructed......406
Eustachius, monument.....486
Emphysema ..............460
Encysted tumor of neck....598

F

Fahs, Dr. Charles F., death of ....................672
February number .........611
Fever, origin and nature—243, 303
French bougies in stricture...682
Fibroid, subserous.........541
Flint's Practice ...........235
Forceps, curious accident...475
Foreign Correspondence—105, 381, 385, 465
Ford, D'Saussure, M.D .....276
Ford, W. H. M.D .........376

G

Glass for dissecting tubes...172
Galaxy, The...............608
Galvan's Therapeutics......731
Gastrotomy for obstruction..587
German schools of Medicine 476
Gellatinous pregnancy.....551
Georgia State Medical Association..............117, 732
Green papeɞ poisoning.....700
Gilmore, J. T., M.D.........175
Griggs, A. W., M.D.........191
Griffith's Formulary.......664
Goat's milk for babes......520
Gonorrhœa of rectum......289
Goodell, Dr. Wm...........475
Gross, Prof. S. D..........454
Guaiac Pills for syphilis....593
Guild, James, M.D .........611

H

Hammond. D. W., M.D....147
Harland, Geo. C., M.D.....386
Harvey, anecdote of.......457
Hair destroyer, new.......591
Hæmatocele, pelvic.........541
Hæmostatic, new..........690
Head-ache, sick...........651
Herald, Atlanta Daily......544
Herald of Health, Wilson's..731
Hernia, radical cure.........520
Herpes zoster, ophthal.....188
Hicks, J. Braxton, M.D....363
Hints to young practitioners 91
Hip, bony auchylosis.......653
Holmes. G. W., M.D....... 22
Hoyt, W. D., M.D..........138
Horsford's Acid Phosphate...178
Homocultology.............542
Hydrochlorate ammonia....214
Hymen intact, delivery.....650
Hyoscyamine ..............240
Hypodermic morphia—134, 604, 646, 668
Hypospadias, operation....587

I

International Medical Congress..............118, 398
Intestinal obstruction......587
Inversion uterus, chronic...474
Iodide potass in asthma....134
Iodoform solution..........528
Iron, admin. perchlor......402
Introductory lecture.......428

J

January number...........547
Jelks, J. T., M.D..........145
Jones, Jos., M.D....157, 280, 468
Johnson, J. T., M.D........ 27
June number..............123
July number ..............183

L

Larynx, syphilis of.........656
Laryngeal phthisis.........457
Laxatives.................460
Laryngoscopy.............469
Lead in the excretion......506
Liebig, death of...........241
Leishman's Midwifery......726
Life, definition of .........583
Life supported by injections..634
Liquor bismuthi ..........529
Logan, Samuel, M.D.......376
Logan, J. P., M.D.........477
London Lancet...........449
Lung cavities, treatment....596

M

Malarial engorgement lung..141
Malarial fever, diarrhœa...687
March number.............675
May number.............. 65
Mamma succenturiata......472
Marsh plant, malarial......560
Medical charity, question...460

Medical Examiner.........524
Medical experts...........298
Medical organization.......113
Medical Record...457, 521, 640
Medical & Surgical Reporter
476, 540
Medical Times........453, 706
Medico-legal questions.....340
Membranes, premature rup.535
Memphis, deceased brethren 673
Menstruation, physiology—
394, 468
Mercury oleate ...........449
Mercury, therapeutics...265, 325
Mercy of prompt surgery...507
Metrorhagia, nit. mercury..639
Midwifery, Leishman's.....726
Minutes Georgia Medical Association................ 40
Mistaking spontaneous recoveries.................179
Mitral obstruction..........540
Monument to Eustachius...486
Moore, K. P., M.D.....214, 317
Moorman on min'rl springs...235
Morse, D. A., M.D.........340

N

Napoleon III, case of......195
Nature and Art.......179, 323
Nebraska State Med. Soc'y..601
Neck, encysted tumor......598
Needle removed from heart.122
Nelaton, death of .........339
Nerve tissue..............553
Nervous system...........611
New Orleans..............290
N. O. Medical and Surgical Journal.................179
Night in the mountains....294
Night sweats..............459
Nitrate Mercury, Metrorhagia...................639
Nitrate silver to uterine cavity ..................121
Nitric acid in whooping cough ..................181
Normal Ovariotomy..1, 65, 175,
231, 732
Normal action medicines...232
Norris' Surgery............408
N. W. Medical and Surgical Journal .................644
Nostrum quackery.........300
Nottingham, C. B., M.D.,—
151, 547
November number.........419
Nurses, hints for..........659
Nursing profession........400
Nutritive injections........657

O

Opaque optic fibres in retina.105
Ovaritis, sub-acute.........532
Ovariotomy ..........151, 454
Ovariotomy, Normal..1, 65
175, 231
Ovariotomy by enucleation .376
Ovariotomy, pysiology double ....................394
Œdema of Scrotum and penis ....................200
Oleate Mercury...........449
Opium cure, case..........703
Opium vs. Belladonna......240
Origin, nature, treat, fever..243
Our New Associates....... 58
Our Exchanges....446, 521
579, 640, 704
Ozonizing sick room.......452
Obstetrical Journal G. B. & I.
470, 529
Obstetrical Journal........179
Obstetric Medicine ........363
October number...........303

P

Patrons Atlanta M. & S. Journal ................403
Pancreatin, note on .......528
Practice at St. Luke's Hosp.463
Placenta prævia...........646
Plantation, The...........545
Peritonitis................459
Persimmon coffee .........560
Peristaltic arterial action...652
President's address........ 22
Pregnancy, diagnosis......473
Pregnancy in the aged.....494
Pregnancy, case abnormal..551

Pneumonia................459
Puerperal Septicæmia......482
Puerperal Diseases, Barker.731
Precocity, extraordinary—
476, 591
Pirogoff's operation........595
Phthisis, laryngeal.........457
Poly-pharmacy............524
Procidentia Uteri..........112
Prostate, catheters in hypertrophy..................579
Professional secrets........585
Poisoning, green paper.....700
Pulmonary tuberculosis....191
Phytolacca Decandria......448

R

Rauchenberg, Ch., M.D.—
92, 205, 243, 303, 419
Ramsey, Dr. Frank A......120
Ralls, John P., M.D.......186
Rank of Med. Corps U. S. A.482
Rattlesnake, rattles........590
Report of cases...........138
Rectum, Allingham on.....416
Rectum, extirpation.......456
Rectum, gonorrhœa of.....289
Resemblance, parents to children...............464
Registration Law.....542, 547
Reynolds on electricity.....731
Richmond and Louisville Medical Journal.........587
Rockwell, A. D., M.D......123
Rockwell and Beard, electro-surgery................607
Rodgers, James, M.D......220

S

Saccharated Calomel—
261, 407, 605
Salutatory...............478
Savannah Medical College..230
Strange suggestion........546
Shaffer, A. J., M.D........551
Siamese Twins, autopsy of..706
Siamese Twins, death of....670
Siamese Twins, history of..723
Senex.................... 91
September number........303
Spleen, removal...........590
Selma mortuary statistics...668
Sick headaches.............651
Simpson, C. A., M. D......442
Skin grafting..............446
Spinal cord, division.......578
Stimulants, craving for....592
Stricture, French bougies...682
Scoptzi in Roumania.......483
Stout, S. H., M.D.........413
Spontaneous disloc. clavicle.655
Subcutaneous morphia—
134, 604, 646, 668
Surgery, Norris'...........408
Surgeon General's Report..481
Surgery, mercy of prompt..507
Sussdorff, G. E., M.D..155, 183
Syphilis, guaiac pills.......593
Syphilis of larynx.........656
Syphilitic infection, sources.704

T

Tape worm, treatment.....462
Tar water, new process....528
Tracheotomy.........119, 155
Transmissi'n of resemblance.464
Transfusion of blood..529, 532
Tea drunkards.............227
Teeth, replanting..........449
Therapeutics of merc'y.265, 325
Triplets, case.........407, 672
Trismas and tetanus.......233
Todd, J. S., M.D..........134
Toner's Dictionary of Elevations..................479
Thompson, J. Harry, M.D..661
Typho-malarial fever......140

U

Uterine fibroid............ 56
Ulcers, treatment..........453
Urination, painful.........546

V

Vaccination, damages for...461
Vaginal injection, death....651
Valedictory...............477
Vanity of Sawbones........593
Ventilation and warming...122
Vertebræ, dislocation......589
Vesico-vaginal fistula......234

W

Walker, John E., M.D.....619
Wall, John T., M.D........682
Walton on Min. Springs....236
Warren, Edward, M.D.....385
Water, purification........265
Wharton trial.............298
What is Cincho-quinine 395, 622
Westmoreland's Acology...413
Westmoreland, J. G., M.D...84--
Westmoreland, W. F., M.D...231
Western Lancet.......465, 650
Wilson's Herald of Health..731
Whitaker, A. S., M.D......407
Whittaker, Jas. T., M.D...495
Women as Doctors.........494
Woodward, J. J., Asst. Surg. U. S. A..................660
Whooping-cough specific...544
Wythe's Dose Book.........731

Y

Yellow Fever.....157, 280, 468

### ACKNOWLEDGMENTS.

Atlanta Herald, Daily; Rome Commercial, Daily; Medical Examiner; American Practitioner; Nashville Journal of Medicine and Surgery; American Journal of Pharmacy; Boston Medical and Surgical Journal; Druggist's Circular; Detroit Review of Medicine and Pharmacy; Richmond and Louisville Medical Journal; Wilson's Herald of Health; Medical and Surgical Reports; Medical News and Library; The Clinic; St. Louis Medical and Surgical Journal; Buffalo Medical and Surgical Journal; Philadelphia Medical Times; Half-Yearly Abstract of Medical Sciences; Western Lancet; London Lancet; Galaxy for March; Chicago Journal of Nervous and Mental Diseases; Popular Science Monthly; Medical Record; Pacific Medical and Surgical Journal; Indiana Journal of Medicine; Herald of Health; Journal of Materia Medica; Contributions to the Natural History of specific Yellow-fever. By Joseph Jones, M.D.; Changes of Temperature and Pulse in Yellow-fever. By Joseph Jones, M.D; Temperature in Yellow-fever. By Joseph Jones, M.D; Yellow-fever Epidemic of Memphis, Tennessee; Transactions South Carolina Medical Association; Transactions Indiana State Medical Society; Transactions Alabama State Medical Association.

ATLANTA MEDICAL AND SURGICAL JOURNAL ................................. 57

OUR NEW ASSOCIATES ............................................... 58

☞ *The Editors are not responsible for the views of Contributors.*

